# Hydra: software for tailored processing of H/D exchange data from MS or tandem MS analyses

**DOI:** 10.1186/1471-2105-10-162

**Published:** 2009-05-27

**Authors:** Gordon W Slysz, Charles AH Baker, Benjamin M Bozsa, Anthony Dang, Andrew J Percy, Melissa Bennett, David C Schriemer

**Affiliations:** 1 Department of Biochemistry and Molecular Biology, University of Calgary, Calgary, Alberta, T2N 4N1, Canada

## Abstract

**Background:**

Hydrogen/deuterium exchange mass spectrometry (H/DX-MS) experiments implemented to characterize protein interaction and protein folding generate large quantities of data. Organizing, processing and visualizing data requires an automated solution, particularly when accommodating new tandem mass spectrometry modes for H/DX measurement. We sought to develop software that offers flexibility in defining workflows so as to support exploratory treatments of H/DX-MS data, with a particular focus on the analysis of very large protein systems and the mining of tandem mass spectrometry data.

**Results:**

We present a software package ("Hydra") that supports both traditional and exploratory treatments of H/DX-MS data. Hydra's software architecture tolerates flexible data analysis procedures by allowing the addition of new algorithms without significant change to the underlying code base. Convenient user interfaces ease the organization of raw data files and input of peptide data. After executing a user-defined workflow, extracted deuterium incorporation values can be visualized in tabular and graphical formats. Hydra also automates the extraction and visualization of deuterium distribution values. Manual validation and assessment of results is aided by an interface that aligns extracted ion chromatograms and mass spectra, while providing a means of rapidly reprocessing the data following manual adjustment. A unique feature of Hydra is the automated processing of tandem mass spectrometry data, demonstrated on a large test data set in which 40,000 deuterium incorporation values were extracted from replicate analysis of approximately 1000 fragment ions in one hour using a typical PC.

**Conclusion:**

The customizable workflows and user-friendly interfaces of Hydra removes a significant bottleneck in processing and visualizing H/DX-MS data and helps the researcher spend more time executing new experiments and interpreting results. This increased efficiency will encourage the analysis of larger protein systems. The ability to accommodate the tandem MS dimension supports alternative data collection and analysis strategies, as well as higher resolution localization of deuteration where permitted by the fragmentation mechanism.

## Background

Hydrogen-deuterium exchange mass spectrometry (H/DX-MS) is a powerful method for relaying information on protein dynamics and protein-ligand interactions. In H/DX-MS, proteins are deuterium-labeled by simple incubation of the protein or protein mixture with D_2_O, which exchanges labile hydrogen with deuterium. The degree of labeling is determined by the rate of exchange, which in turn is determined by the structure and dynamics of the protein. These exchange rates are influenced by the binding interactions of the protein. If exchangeable hydrogens are involved in the ligand binding site, these will be 'less available' for exchange and will exchange at a lower rate than in the absence of the ligand [1]. While N-terminal and most side-chain hydrogens exchange very quickly and escape detection, amide hydrogens exchange in a time scale supporting measurement at a temporal resolution approaching a few milliseconds [2]. The overall workflow for typical bottom-up H/DX-MS experiments has been reviewed ([3] and [4]).

The readout for H/DX-MS is the mass spectrum. In a bottom-up H/DX-MS approach, H/D exchange is monitored at the peptide level and the deuterium-labeled peptide generates an isotopic envelope which is well modeled by binomial expansion(s) of the underlying unlabeled counterpart [5,6]. Extraction of deuterium levels typically requires extraction of the envelope for each peptide of each raw data file. H/DX-MS studies can consist of a set of experiments that monitor tens to hundreds of peptides. Often two or more states of a protein require comparison (for example, free vs. bound or phosphorylated vs. unphosphorylated). Proteins may be labeled for durations of msec's to days in order to generate kinetic data [7]. Varying the concentration of D_2_O used in the labeling reaction may also be required [8]. Thus, while individual H/DX-MS experiments can be completed rather quickly, thorough protein analysis usually involves numerous runs with the attendant requirement for replicates for each run. This creates a burden in data analysis, where tens to hundreds of LC-MS files require inspection. A set of experiments for small proteins can take several weeks to analyze, if processed manually.

Several tools have been developed to address MS-level data processing needs. HX-Express is a semi-automated Excel-based application that extracts average deuterium incorporation levels from the user-supplied experimental and theoretical isotopic distributions. CalcDeut [9] and DEX [10] are software packages that extract deuterium incorporation distributions from user-provided mass spectra, through a command-line approach. These tools require manual processing of extracted ion chromatograms (XIC's) and mass spectra and do not attempt to provide a means of grouping raw data inputs or organizing and aggregating data outputs on a project-wide scale. This is also the case for AutoHD, an earlier approach to deuterium incorporation measurement [6].

**Figure 1:**
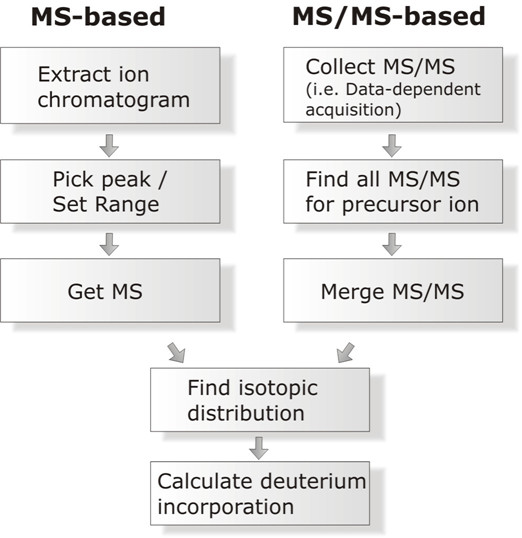
**H/DX-MS and HDX-MS/MS data processing**.

Two packages are available that operate on a project-wide scale. The Deuterator [11], is a freely available web-based tool that organizes the raw data files according to experimental conditions, and effectively completes all the tasks in the MS-based workflow shown in Fig. 1. As with AutoHD, It uses a 'theoretical fit method' to measure deuterium incorporation, in which the theoretical isotopic profile for varying degrees of deuterium labeling is fit to the experimental isotopic profile. Deuterium incorporation values can be outputted and further data handling is left to the researcher. A newer version, 'HD Desktop' [12], provides additional data handling capabilities. 'TOF2H' is a second package [13]. This is a recently published Excel-based application geared toward processing and viewing MS-level MALDI-TOF data from the AB 4700 TOF-TOF instrument. TOF2H organizes raw files, and iterates through the typical processing workflow shown on the left-hand side of Fig. 1. Other packages have been described in the literature, but are not as readily available [14,15].

Though the Deuterator and TOF2H greatly advance an automated approach to H/DX-MS data processing, they do not meet several of our data analysis requirements. We required a customizable data analysis workflow in order to evaluate novel H/DX-MS procedures such as the one we recently reported, involving restraints on the peak envelope width [8]. This demanded an architecture supporting easy adaptation. We were also interested in automating deuterium distribution calculations, methods that were described by several groups including ours [6,9,10,16,17] but not incorporated into a project-wide scale with a convenient graphical interface. Further, as our group conducts large scale studies on large multiprotein systems, retaining file size efficiency was an important operational requirement. Most of our experiments are conducted on QSTAR instruments thus we needed a utility for processing Analyst QS .wiff files. Avoiding a file conversion process has advantages in time savings. While .wiff files could be converted to mzXML for use in the Deuterator, the conversion greatly increases file sizes and these projects would require uploading to the Deuterator FTP site, which could be cumbersome for large projects. We recognize that retaining platform independence is a useful attribute, thus the software also required a means of processing mzXML files efficiently.

Most importantly, we required an automated approach to the extraction of deuteration data from MS/MS spectra on a large scale, such as might be generated in a proteomics-style approach to H/DX experiments. Although extracting deuteration data from MS/MS spectra is not yet common, two recent observations argue for its routine implementation. First, we have demonstrated that deuterium scrambling, common in collisionally-induced dissociation (CID) of peptides, enables robust measurements of deuteration reflective of the precursor ion (manuscript in preparation). Second, recent studies have highlighted that alternative fragmentation processes such as electron capture dissociation may suppress scrambling and support higher resolution localization of deuteration [18]. Both will require new software for automated processing of data. The right-side of Fig. 1 highlights the additional considerations that such a software package needs to address, for data collected in a conventional proteomics fashion (i.e., data-dependent acquisition with possible "include ion" lists). This article will describe how Hydra meets each of these new requirements, in addition to providing features available in other H/DX-MS data processing packages.

## Implementation

Hydra was written in C# (.NET Framework 3.5). A stand-alone executable setup file facilitates installation on the target computer. Hydra allows processing of AB/Sciex QSTAR data (.wiff files) directly, which requires a local installation of Analyst QS 1.1 (AB/Sciex, Foster City, CA). An alternative version processes mzXML files after a file conversion step, and does not require Analyst QS.

Generation of extracted ion chromatograms (XIC's) and MS spectra from Analyst QS files uses libraries available within this software package. Hydra leverages several additional publicly available class libraries. All graphing controls are from Zedgraph version 5.1.4 [19]. For platform independent mzXML files, XIC's and MS spectra were generated using class libraries from ProteoWizard [20] and Decon2LS [21] was used for peak detection and Savitzky-Golay smoothing. For both versions of Hydra, Lutz Roeder's C# library 'Mapack' [22] was used for performing linear least squares operations for the extraction of deuterium incorporation distributions. Numbers generated by this library were tested for agreement with values generated from the command-line program 'CalcDist.py,' as described in Chik *et. al *[9]. Molecular weight calculations and theoretical isotopic distributions were calculated using the class library 'MwtWinDll.dll' (version 3.1) provided as part of source code for Pacific Northwest National Laboratory's Molecular Weight Calculator [23]. Calculation of Student's t-values and P-values were carried out using C# source code from the ALGLIB project [24]. All the class libraries listed above are included in the setup file for Hydra.

Unit testing using NUnit [25,26] was performed throughout Hydra to ensure well-functioning source code and algorithms that return appropriate values.

## Results and discussion

**Figure 2:**
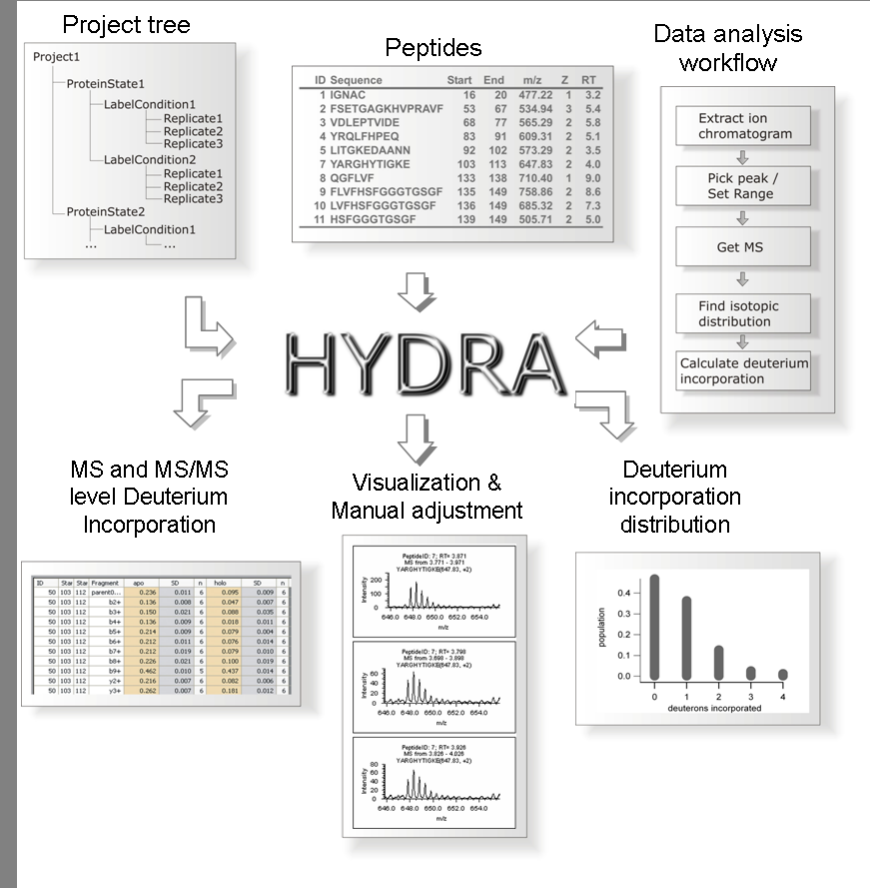
**Key inputs and outputs of Hydra**.

An overview of Hydra is provided in Fig. 2. The main outputs include 1) deuteration values at both the MS and MS/MS level 2) data visualization for manual assessment and validation and 3) distributions of deuterium incorporation. These are described in detail below. One of the key design features of Hydra relates to the configurability of the workflow. This will be described in conjunction with the automated processing capabilities of Hydra.

### Automated extraction of deuteration values and flexible workflows

**Figure 3:**
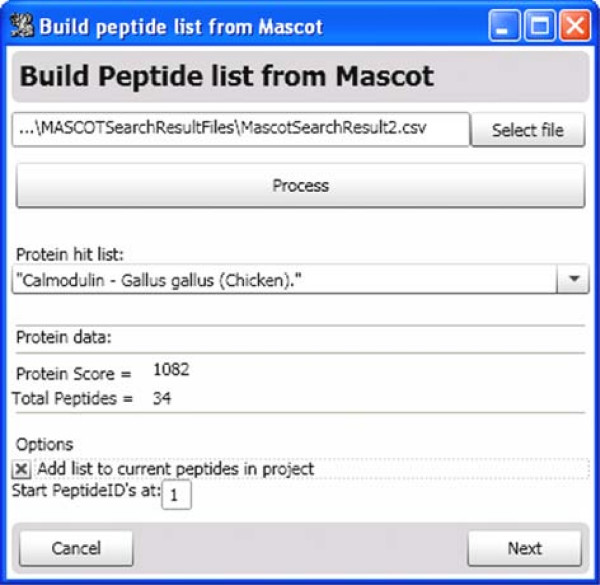
**Inputting Peptide Data from a MASCOT search result**. After selecting a Mascot search result exported in 'csv' format, the user clicks 'Process'. Hydra parses the search result and lists the protein hits. The peptide data (*m/z*, retention time, charge state, sequence, etc.) from the selected protein are imported into Hydra.

Hydra focuses on bottom-up H/DX-MS experiments and therefore examines deuteration incorporation at the peptide level. Minimally the researcher must supply the sequence, *m/z*, expected retention time, and charge state for each peptide. This can be accomplished within Hydra by parsing the Comma Separated Values (.csv) output of a Mascot search result, for example, allowing a quick way to import peptide data from a common database search program; a screenshot of this tool is shown in Fig. 3. Alternatively, the peptide data may be entered manually within Hydra or from a user-generated .csv format (i.e., using Excel.

**Figure 4:**
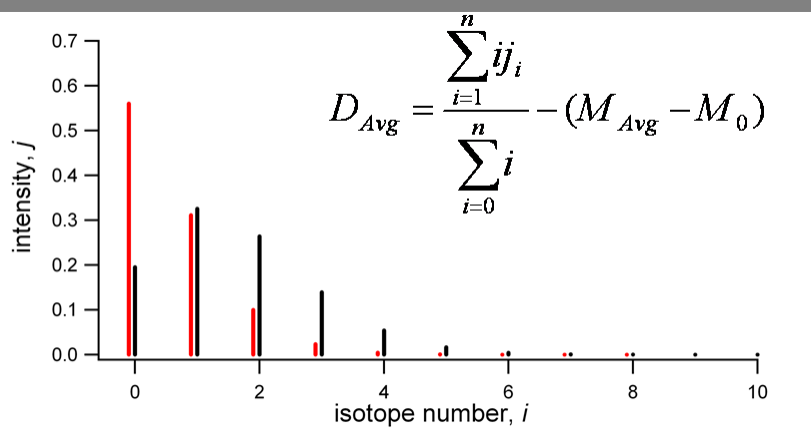
**Extracting deuterium levels from MS data**. The expansion of a native isotopic distribution for a peptide (red) resulting from deuteration (black) can be quantified in terms of an average deuteration level (D_avg_), as shown in the equation (inset). Here, M_avg _refers to the average mass of the peptide ion and M_0 _the exact mass of the peptide monoisotopic ion.

On a basic level, several steps are required prior to extraction of the deuteration data. The first is extraction of peptide ion chromatograms (XIC's). This reveals the liquid chromatographic elution profiles for the peptides. In a typical experiment, several peptides may share the same *m/z *as the target peptides, thus the XIC's often contain several peaks. A peak is usually selected based on the expected retention time for the peptide within a given tolerance or via a specified time-range. Integration over the XIC peak or specified range generates the mass spectrum. The mass spectrum contains many peaks and the isotopic profile for the target peptide must then be located. The deuteration level can then be calculated in a number of ways. The most common approach is the 'centroid method' in which the average mass of the isotopic profile is calculated, either using isotopic peak intensities and *m/z *values, or all *m/z *and intensity data points within a defined *m/z *range. Deuterium incorporation can be calculated by a simple formula (see [8]) using the experimental centroided mass and the theoretical centroided mass, which may be calculated from the theoretical isotopic profile of an unlabeled peptide. This 'centroid method' is highlighted in Fig. 4, showing that the intensities of the individual isotopic peaks are required for such measurements. Other methods of calculating deuterium incorporation are discussed later in the text. Hydra provides a tool for global adjustment of most peptide data fields. This is useful for quickly adjusting fields that are often the same for each peptide (i.e. XIC integration width, threshold levels, etc).

Fig. 2 summarizes the three basic inputs into Hydra for automation of data extraction for multiple datasets: 1) a project tree, which functions to associate raw data files with experimental conditions; 2) the peptide set and 3) the data analysis workflow. The project tree is a means of organizing all the raw data files within a confined set of experiments. As an example, we have studied the calcium-free (*apo*) vs. the calcium-loaded (*holo*) forms of the protein calmodulin under six different D_2_O concentrations (5–50%), with the labeling time held at two minutes. We generated six replicates for each concentration. Additional file 1 shows Hydra's project setup interface that was used in defining this project. In this example, the project tree was populated with the raw data files according to the two 'protein states' – *apo *and *holo *– and the six labeling conditions with replicates. Currently, Hydra allows processing of both AB/Sciex .wiff files and generic mzXML files. Future versions of Hydra will implement new generation mzML files.

**Figure 5:**
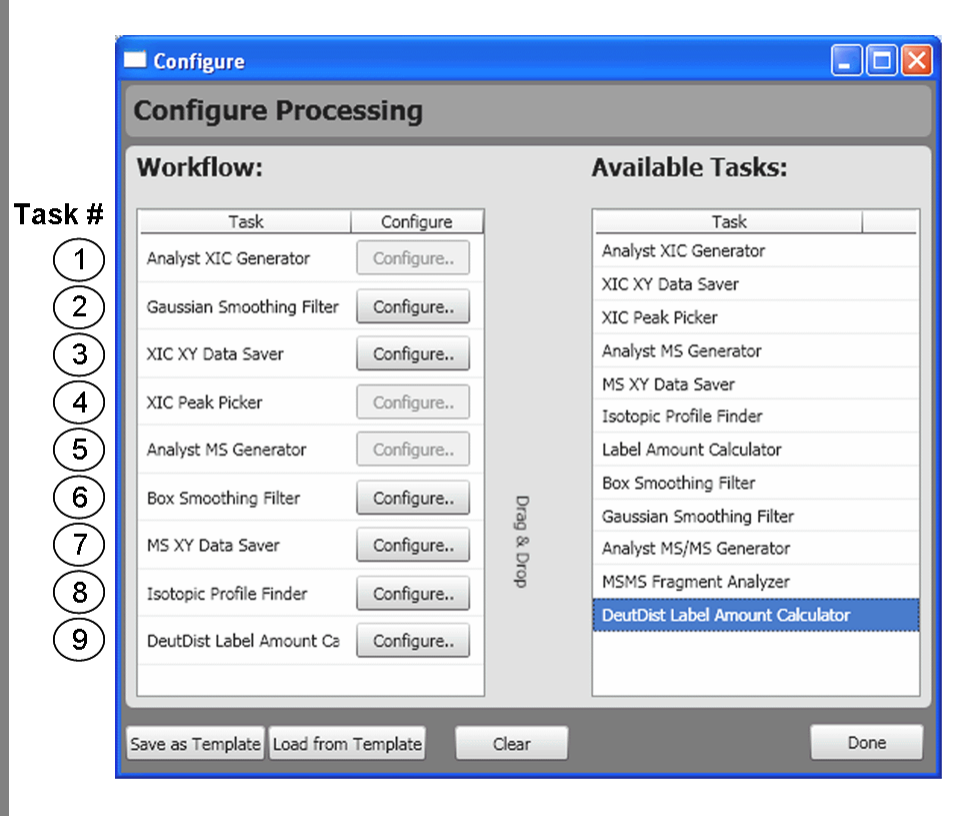
**Configuration of the data processing workflow**. All available data processing tasks currently available are listed (right-hand side). These are dragged-and-dropped into the data analysis workflow (left-hand side). Several of the tasks can be individually configured. Hydra verifies the workflow and notifies the user if problems with the workflow are detected (e.g. missing tasks or nonsense ordering). Saved workflows can be retrieved. A sample workflow is shown in the screenshot and the numbered tasks are referenced in the text.

Once the peptide set and the project tree have been defined, the data processing workflow must be established. Rather than force a given workflow on the process, we have enabled easy customization and configuration of data analysis to facilitate the addition of new processing algorithms into the software code base (e.g. new peak detection algorithms). Fig. 5 shows a screenshot of the Workflow Configuration Window. The available algorithms (or 'tasks') are displayed on the right-hand side. These are 'dragged-and-dropped' into the data analysis workflow and can be clicked to reveal underlying customizations. The left-side of Fig. 5 shows a typical H/DX-MS workflow that firsts extracts an ion chromatogram, provides smoothing, selects the appropriate peak, integrates over the peak to give the mass spectrum, provides smoothing and peak detection, locates the isotopic profile, and finally calculates the deuteration level.

The modular nature of the workflow set-up allows flexibility in the tasks that are selected, how they are implemented, and in what order they are performed. At the software level, this is made possible through the dynamic loading of algorithm assemblies that implement a plugin interface defined by Hydra. Each algorithm is derived from an abstract 'task' that simply requires one input (the Result object) and has one function ('DoTask'). Each task is executed and thus populates the Result object. Configurations are embedded within each task and the workflow is saved as part of the entire Hydra project, allowing easy retrieval of previous workflow settings. The modular nature of the workflow allows a great deal of freedom in defining workflows. For example, in this first release of Hydra, we provide two alternatives for calculating average deuteration levels. One provides the conventional method of measuring the centroid mass of the isotopic distribution, while the other extracts the underlying deuterium distribution and uses this data to calculate the labeling level [9] (see below for further description of implementation of deuterium distribution calculations in Hydra).

Although the current algorithm toolbox (see Fig. 5) limits the user to one or two choices per algorithm, we envision the addition of algorithms previously described by others, especially in the area of peak detection, isotopic profile isolation, and label amount calculation. OpenMS has recently been released and offers robust MS feature detection [27] and if implemented into Hydra, will provide a strong peak-finding alternative. In Hydra, we use a simple algorithm for locating an isotopic profile, resembling that described by Pascal et al. [11]. In the Hydra algorithm, the monoisotopic peak is first located; then other members of the isotopic profile are populated by searching within a *m/z *window for peaks that match the expected *m/z *and charge state of the peptide. While this simple algorithm has been used successfully by our lab for over two years, we recognize that it is less successful with convoluted mass spectra. Use of more complex algorithms such as THRASH [28] or NITPIK [29] will be extremely beneficial for teasing apart overlapping peptides. As noted above, Hydra provides two alternatives for deuterium incorporation calculation. Using a isotopic distribution fitting algorithm to calculate label incorporation such as the method used by the Deuterator [11] or most recently described by Sperling *et al*. [30] will be useful additions. Hydra's framework should speed the implementation of these and other algorithms. We note here that Hydra was primarily designed for high resolution MS data, where baseline resolution of isotopic peaks within an envelope promotes greater accuracy and precision in deuterium level calculations.

The most obvious benefit to automatic H/DX-MS data processing is speed of analysis. To examine this, the workflow shown in Fig. 5 was applied to a set of experiments involving tubulin conformation analysis, first using the Analyst-based version of Hydra. Tubulin is a relatively large dimeric protein (110 kDa) that yields a complex mixture of peptides challenging to many facets of H/DX-MS data processing, validation, and visualization. We have identified over 400 peptic peptides, about 200 of which we monitor during data analysis. Since the peptide mixture is complex, XIC's of the target *m/z *values often feature several elution peaks and mass spectra are frequently convoluted with overlapping peptides. In this test project we compared two biochemical states of tubulin, each state consisting of 1 labeling condition (50% D_2_O), with a total of 9 raw data files. Deuterium incorporation data was extracted for 189 tubulin peptides from Analyst™ QS 1.1 '.wiff' files. Data processing in this project required the evaluation of 1701 mass spectra; Table 1 displays the analysis times. To test the processing of mzXML files using the Analyst-based version, mzXML files were generated from '.wiff' files using Seattle Proteome Center's mzWiff.exe command-line application [31]. As Table 1 shows, processing raw data was nearly twice as fast as processing mzXML files, demonstrating the efficiency of direct file access. The time difference is tolerable in most situations, since a first-pass extraction of the data is normally performed once. Clearly, processing mzXML files using the Analyst-based version of Hydra is awkward when using data from other instruments, as a license for Analyst is required. Thus we developed an Analyst-free version where Analyst algorithms were replaced with publicly available libraries. This version offers a comparable efficiency to the Analyst version for processing mzXML files (data not shown).

**Table 1:** Processing and reprocessing times for the tubulin H/DX-MS project

	First-pass Processing	Reprocessing	
		
	Tasks 1–9* (seconds)	Tasks 4–9 (seconds)	Task 9 (seconds)
Analyst™ raw data	1909/1.1**	620/0.4	0.9/0.0005
mzXML data	3189/1.9	1422/0.8	7.0/0.0042

* Refers to tasks listed in Fig. 5.

** Processing time for entire project/processing time per peptide

Table 1 also reports the time required for reprocessing the data. The ability to reprocess data at various starting points is a direct benefit of the workflow design, described above. Once first-pass processing is complete, the result object can be shuttled through the algorithms. This results in a substantial time benefit when testing various data processing schemes or making adjustments to settings within a particular task in the workflow. One can envision reprocessing the data with an alternate algorithm for calculating deuterium incorporation without having to re-generate and re-locate the isotopic profile. In the test case, re-executing the label amount calculation required only 1 second for the entire project. Reprocessing is an important aspect of our manual assessment and validation tool, described below.

Once processing is complete, the project can be saved in a binary format and can be reloaded at a later time. Hydra allows some control as to what data is saved. For most projects it is convenient to save all chromatograms, mass spectra and deuterium incorporation data for all peptides. For the tubulin test project, the Hydra file size for both the Analyst-based and mzXML-based projects was around 40 Mb. To avoid extremely large file sizes for even larger protein systems, the user can remove the 'data saver' task from the workflow. In this case, the individual chromatograms and/or mass spectra will not be saved, although this will come at the cost of slower re-processing.

### Viewing, validating, and re-processing tools

**Figure 6:**
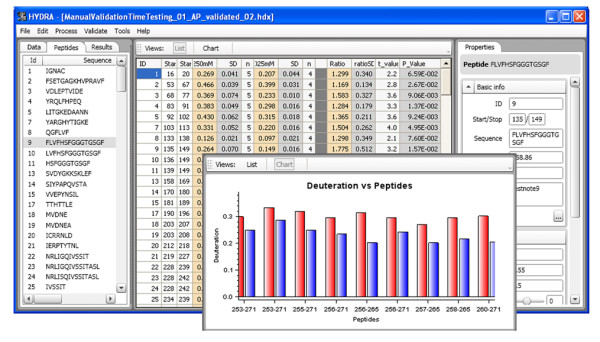
**Primary layout of Hydra for viewing data output**. The example provides a table of the averaged deuterium incorporation values for two protein states, along with and other statistical measures. The screen is divided into three areas. The left-most area is used for navigating: the 'data' tab shows the project tree, the 'peptides' tab lists the peptide data set and the 'results' tab allows the user to navigate various views of the data. The center displays results in either tabular of graphical form, where the inset shows a 'chart' view of deuteration values for the two protein states (red and blue), zoomed into particular range of peptides. The right-most area shows detailed context-sensitive properties.

Fig. 6 shows the primary layout of Hydra as it is used in viewing the processed data. The screen is divided into three sections. The left-most section is used for navigating through the project tree, including protein states, labeling conditions and individual peptides. The center area displays graphs and tables of data and the right-most section presents context-sensitive properties of the selected feature of the project tree. The primary means of viewing deuterium incorporation data is through the summary table, shown in the center section of Fig. 6. This table reports the averaged deuterium incorporation values and their standard deviations for the replicates for each peptide of each protein state. In the sample table of Fig. 6, we compare the *apo *versus *holo *forms of calmodulin. Comparative statistics are shown in the right-most columns of the table. These include the deuteration ratio and its standard deviation, a Student's t-test and P-value for detecting differences between the two protein states. Right-clicking on the table allows the user to control the selection of protein states being compared. A graphical view of the data is also shown in the screenshot overlaying the table.

**Figure 7:**
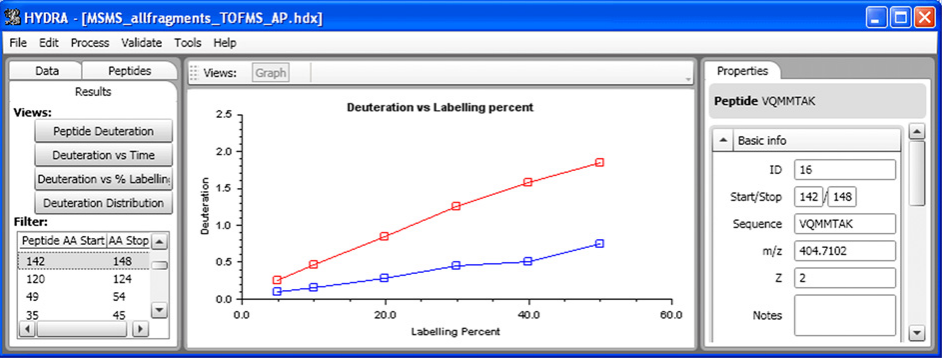
**Viewing peptide-specific data for multiple labeling conditions**. In this particular project, the percent D_2_O used in labeling two protein states (red and blue) was varied. The 'Deuteration vs. % Labelling' view, along with a single peptide, is selected in the 'Results' tab in the navigation pane. The corresponding graph is displayed in the central pane.

Graphical tools are also available for viewing the data as a function of labeling time or labeling percent. In a sample project we examined *apo *and *holo*-calmodulin over a range of D_2_O concentrations (5 – 50%). The center pane of screenshot in Fig. 7 shows the deuteration incorporation measurements for the two protein states for peptide E84-F89 (+1) as a function of labeling percent. Other peptides can be viewed by selection in the left-most pane, and details of the selected peptide can be seen in the right pane.

**Figure 8:**
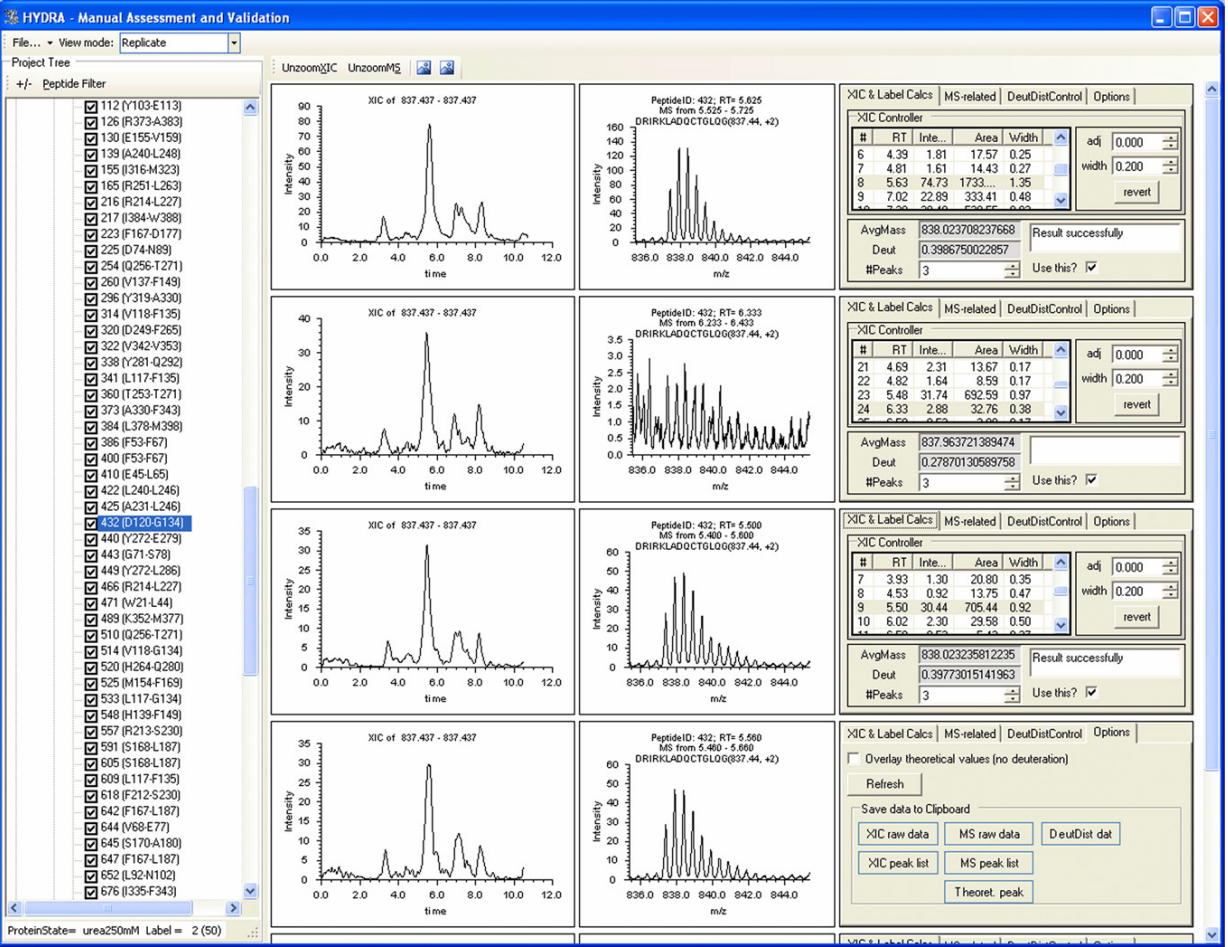
**Manual assessment and validation in Hydra**. The 'Manual assessment and validation' tool allows inspection and adjustment of individual experiments. Shown here is the replicate viewing mode, selected in the top left. XIC's and MS spectra from replicates are vertically aligned, to aid detection of anomalies. In this example, a particular peptide is selected from the project tree on the left. The XICs (left-most graphs) are very similar, while the second MS spectrum is different from the others. The user can then adjust the XIC selection (peak selection, XIC integration width or offset) using the controls in the right-most panel. The file will automatically reprocess as defined in the workflow. The right-most area also contains controls for displaying deuterium distributions, and extracting raw values (see lower-right hand portion of the figure).

Hydra also provides a means for 'drilling down' to allow visual assessment, manual validation and reprocessing of individual replicates. Fig. 8 shows a screenshot of the 'Manual Assessment and Validation' form, whose arrangement resembles that of Hydra's main display: the left-side for navigation, the center for XIC and MS graphs. The right-side contains controls for adjusting the individual replicates (see below). There are three viewing modes: 'quick view' for viewing single replicates; 'replicate view' for vertically displaying all replicates of a given protein state/labeling condition/peptide/fragment (as shown in Fig. 8); and 'Protein state compare view' for vertically comparing data across protein states.

**Figure 9:**
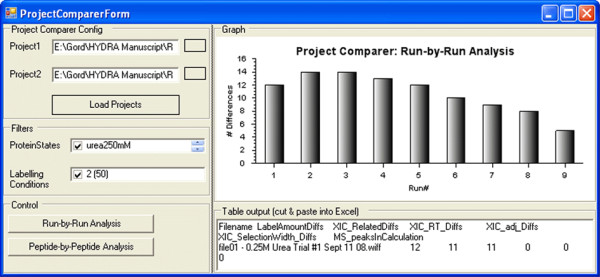
**Comparison of projects before and after manual validation**. The Project-comparer tool is useful for comparing a project before and after adjustments are made. The project is initially saved before any adjustment and then saved again under a different name after manual adjustments are made. Two comparative modes are possible. The 'Run-by-Run Analysis' mode shows differences in the deuteration amount calculation between the two projects, reported for each individual experiment ('Run') in the project. The 'Peptide-by-Peptide Analysis' reports differences between the two projects on a per peptide basis.

The replicate viewing mode is heavily used during manual validation and adjustment of the data and is a key time-saving aspect of Hydra. Vertical alignment of the XICs and mass spectra for all replicates is quick way of visually spotting problems and rejecting spectra (see Fig. 8). The most common problem we encounter in processing MS-level data is in the correct selection of the XIC peak from "busy" chromatograms. Global performance was evaluated using the tubulin test project, in which we used Hydra to automatically process the data and manually validate and adjust the results. Fig. 9 shows a screenshot of the 'Project-comparer tool' that was used to follow the run-by-run number of adjustments that were required for optimum extraction of data from the tubulin test. Comparing the pre- and post- adjustment phase projects showed that adjustments were required for an average of 10.8 of 189 peptides per run, giving a success rate of 94% for automated extraction. The 'Project comparer tool' also reports adjustments on a per peptide level, and helps reveal 'problem peptides.' Hydra's visual alignment of all replicates contributed to the speed of adjustments, which required approximately one hour for the entire project set.

Adjustments of the individual peptide results can be easily made to the XIC peak retention time, the XIC integration window (width and offset), and the number of peaks from the isotopic envelope to be used in the deuteration amount calculation. When an adjustment is made, the individual result is re-processed according to the workflow that was initially defined in the project. Reprocessing is executed only using the tasks necessary. This is done real-time and is nearly instantaneous. For example, in the workflow described above featuring 9 tasks (Fig. 5), an adjustment of XIC peak selection (performed by clicking on a peak in the peak list box) only requires reprocessing using tasks 4 ('MS generator') through 9 ('label amount calculator'). This adjustment required about 0.4 seconds per peptide.

### Deuterium distribution tools

**Figure 10:**
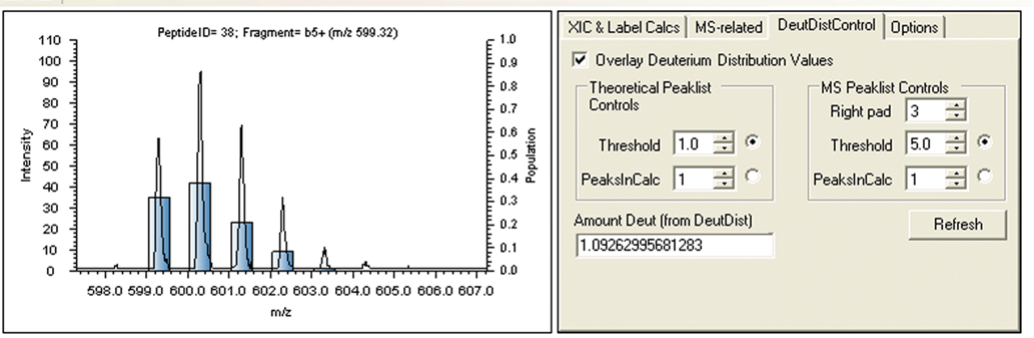
**Deuterium distribution tools**. The deuterium distribution can be extracted using the tools in the "DeutDistControl" tab in the "Manual Assessment and Validation" form (see Fig. 7). The distribution is shown as a blue bar graph overlaying the mass spectrum. Parameters for truncating the theoretical and experimental isotopic envelope are controlled by setting the relative threshold (%) or by setting the exact number of peaks to be used in the calculation (see Chik et. al [9]). A truncated isotopic profile can then be 'padded' using the specified number of zero-intensity peaks. The amount of deuteration can be calculated by the weighted average of the reported distribution and this is displayed within the "DeutDistControl" tab.

Multiple algorithms have been described that extract the deuterium distribution of an isotopic profile [6,9,10,16,17], but none of these have been incorporated on a project-wide scale into a convenient user interface to support rapid visualization of deconvolved isotopic distributions. In Hydra, we implement the method described by Chik *et al*. [9], which uses a linear-least squares approach to fit the deuterium distribution. This can be included as a task within the data processing workflow, and can be further examined in the manual assessment and validation tool, as shown in Fig. 10.

In this deuterium distribution method, the measured isotopic envelope is truncated by excluding peaks whose normalized intensities fall below a user-defined threshold, and the measured isotopic profile is 'padded' with a user-defined number of zero-intensity peaks. In Hydra, deuterium distribution values can be overlaid on the actual mass spectrum (see figure) and the two parameters – threshold and padding – can be adjusted with live-updating. Enabling visualization of the deuterium distribution in this way helps to detect departures from "normal" labeling conditions well modeled by a single binomial distribution. For example, transition from an EX2 to an EX1 exchange phenomenon would be revealed in a departure from a simple binomial distribution of states [32]. It may also support new validation strategies. For example, the deuterium distribution can visually show anomalies in the isotopic envelope that may be missed by visual inspection of the mass spectrum (see Additional file 1). Detection of these problematic spectra through the use of deuterium distribution analysis provides a useful means of automating validation, which remains the bottleneck in data analysis.

### Mining the MS/MS regime

The MS/MS regime in bottom-up H/DX studies is a largely untapped data reserve. Two reasons likely contribute to the underutilization of MS/MS data – one is the perception that CID-based MS/MS data is of little value because of deuterium scrambling. Another is the lack of software to automatically extract deuterium incorporation values from LC-MS/MS spectra, precluding the large-scale analysis of many fragment ions from many peptides. We have demonstrated the analytical utility of the MS/MS domain for deuteration measurements (manuscript in preparation) and recent studies have shown the potential for ECD fragmentation to preserve deuterium location [18]. Thus we developed Hydra to accommodate MS/MS data. Here, we describe the implementation of MS/MS data processing and present some preliminary results. The full evaluation of the MS/MS domain for H/DX-MS studies will be presented elsewhere.

Hydra provides two tasks that enable MS/MS data processing. The 'MS/MS generator' works on both raw Analyst™ QS data and mzXML data to extract the MS/MS spectrum for each peptide precursor ion. This data can be extracted from conventional data-dependent acquisition runs, with or without extensive "inclusion lists". User-controlled parameters define the acceptable *m/z *mass tolerance and the width of a eluted chromatographic peak. These parameters guide the merging of multiple MS/MS spectra that may have been acquired as part of an automated data-dependent MS workflow. The 'MS/MS Fragment Analyzer' is comprised of two tasks – an isotopic profile finder, and the label amount calculator. This Fragment Analyzer takes the MS/MS spectrum, loops over the list of user-provided fragment ions for each peptide analyzed and extracts the deuterium incorporation amount for each fragment ion. Smoothing tasks can be inserted into the data processing workflow as desired.

Multiple workflows can be executed to extract both MS-level and MS/MS-level deuteration incorporation values on the same project. As an example, we examined the deuteration incorporation for the precursor ions and for all singly and double charged b- and y product ions, for 37 calmodulin peptides representing *apo *and *holo *calmodulin under five labeling conditions (5–50% deuterium labeling), with up to six replicates for each condition. This required data extraction from 37 precursor ions and 1002 fragment ions from 70 raw data files. Table 2 summarizes the statistics and time required for extraction of MS- and MS/MS-level deuterium incorporation values for this project. MS-level processing of all peptides in all raw data files required 13.8 minutes or 0.32 seconds per peptide. This is about 4 times faster than the tubulin project described above, primarily because this experiment consisted of both MS- and MS/MS-level spectra whereas the tubulin test project contained only MS-level data. Consequently, generating MS-based XICs for the calmodulin test data set was much quicker than for the tubulin test data since the chromatograms have a reduced sampling rate to accommodate MS/MS spectral acquisition. Indeed, we have found that XIC generation is the most time-intensive task within our typical data processing workflows.

**Table 2:** Statistics for processing MS and MS/MS data

Descriptor	Value
Total raw data files	70
Peptide ions analyzed	37
Fragment ions analyzed	1002
Total 'result objects' generated	40710

MS-level: process entire project (min)	13.8
MS-level: time per peptide (sec)	0.32
MS/MS level: process entire project (min)	63.8
MS/MS level: time per fragment (sec)	0.05

The same project file was re-processed using a MS/MS workflow. In this 'shotgun' data extraction approach, we attempted to extract data for all singly and doubly charged y- and b- fragment ions for all peptides, as well as the precursor ion in the MS/MS spectrum, which amounted to a total of 1002 fragment ions. Total time for executing the MS/MS workflow was 63.8 minutes or 0.05 seconds per fragment ion. Both MS- and MS/MS data can be assessed and evaluated using the assessment and validation tool described above. Additional file 1 displays the 'ProteinState_compare view' for assessing MS/MS spectra. The saved project file was approximately 194 Mb in size. While extracting as much fragment ion data as possible is used here to demonstrate the capability of Hydra to extract large quantities of MS/MS-level values, alternative data analysis strategies could include selective fragment monitoring.

## Conclusion

Hydra accommodates an efficient yet tailored approach to automation of deuteration data from complex sets of bottom-up H/DX-MS experiments. Such automated extraction permits a high success rate when applied to larger protein systems, where a streamlined manual validation utility supports rapid identification and correction of instances where accurate data extraction fails. This automation extends to the deconvolution of the underlying distribution of deuteration states, a powerful tool for studying the underlying exchange process on a global level and supporting a semi-automated error-checking capacity. The design strategy applied to the development of Hydra promotes easy incorporation of additional algorithms to improve facets of the workflow (e.g., peak detection) or to simply adopt alternative strategies. Finally, enabling the extraction of deuteration data from MS/MS spectra collected in a conventional data-dependent fashion will open up a proteomics-style approach to the collection of such data, which is expected to increase the range of application for this technique.

## Availability and requirements

**Project name**: Hydra

**Project home page**: 

**Operating system(s)**: Windows XP with SP2 or SP3; Vista is not supported by Analyst™ QS 1.1. The Analyst-free version is functional on Vista.

**Programming language**: Microsoft C#.NET

**Other requirements**: Microsoft .NET Framework 3.5;

**License**: free software

**Any restrictions to use by non-academics**: none

## Abbreviations

MS: Mass spectrometry; MS/MS: Tandem mass spectrometry; *m/z*: mass-to-charge ratio; H/DX-MS: Hydrogen/deuterium exchange mass spectrometry; XIC: Extracted ion chromatogram; CID: Collisionally-induced dissociation; csv: Comma separated values

## Competing interests

The authors declare that they have no competing interests.

## Authors' contributions

GS provided requirements, developed algorithms and some of the user interface, performed Unit testing and wrote the manuscript. CJB led the project, designed the software architecture, designed and developed the user interfaces and edited the manuscript. BB and AD designed the software architecture and developed core software framework. AJP and MB collected data in support of this project and tested the software. DS initiated and directed the project, provided requirements, tested the software, edited and finalized the manuscript.

## Supplementary Material

Additional file 1 **This section contains additional screenshots of various aspects of Hydra functionality, including project setup, deuterium distribution detection and MS/MS-based data extraction.** Click here for file
