# Supplementary material for: Hydra: software for tailored processing of H/D exchange data from MS or tandem MS analyses
Source: BMC Bioinformatics. 2009 May 27;10:162. doi: 10.1186/1471-2105-10-162 (PMC2696453; doi:10.1186/1471-2105-10-162)
Supplement: Additional file 1 — This section contains additional screenshots of various aspects of Hydra functionality, including project setup, deuterium distribution detection and MS/MS-based data extraction. [file 1471-2105-10-162-S1.pdf]

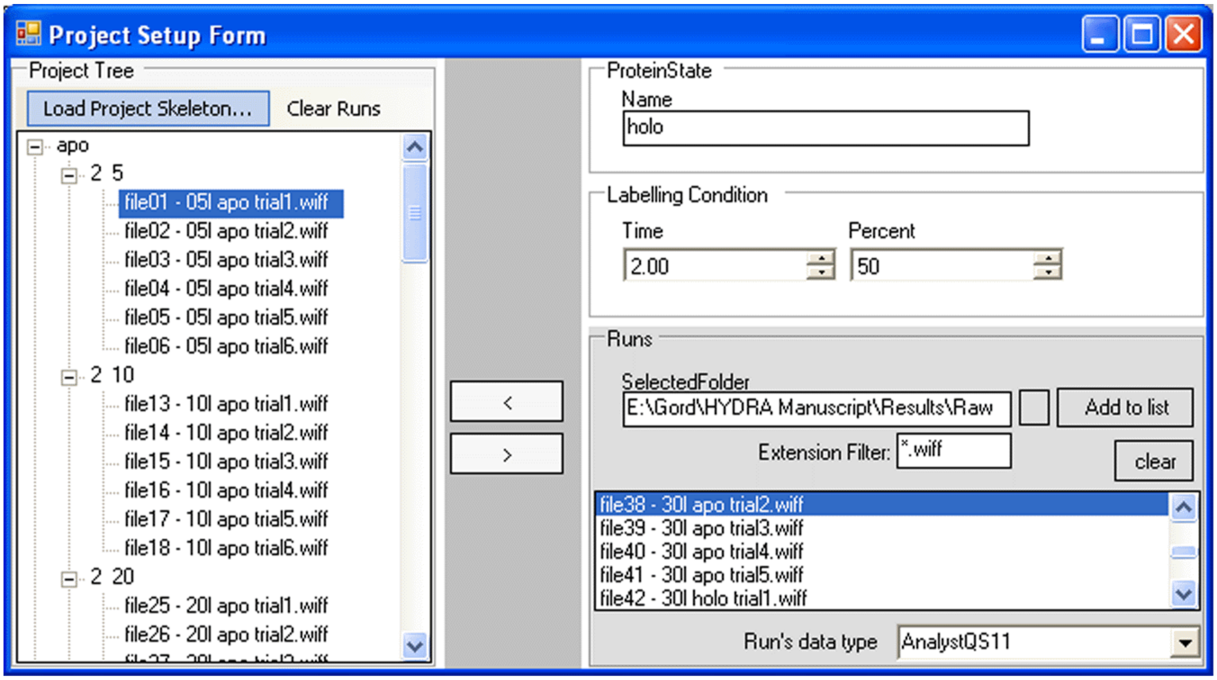

Figure 1 - Hydra's interface for setting up a new project.

The project tree can be quickly assembled using the Project Setup Form. First, the names of the protein states are entered and these are sent over to the Project Tree (on the left-hand side) by clicking the left arrow. Next, the labeling conditions are added and sent over to the Project Tree. Then individual raw data files are associated with their particular protein state and labeling condition. Any branch of the Project Tree can be removed by first selecting it and then clicking the right arrow. Currently, the software operates using either MDS Sciex '.wiff' files or mzXML files and these datatypes are automatically detected by Hydra when the user selects the data file.

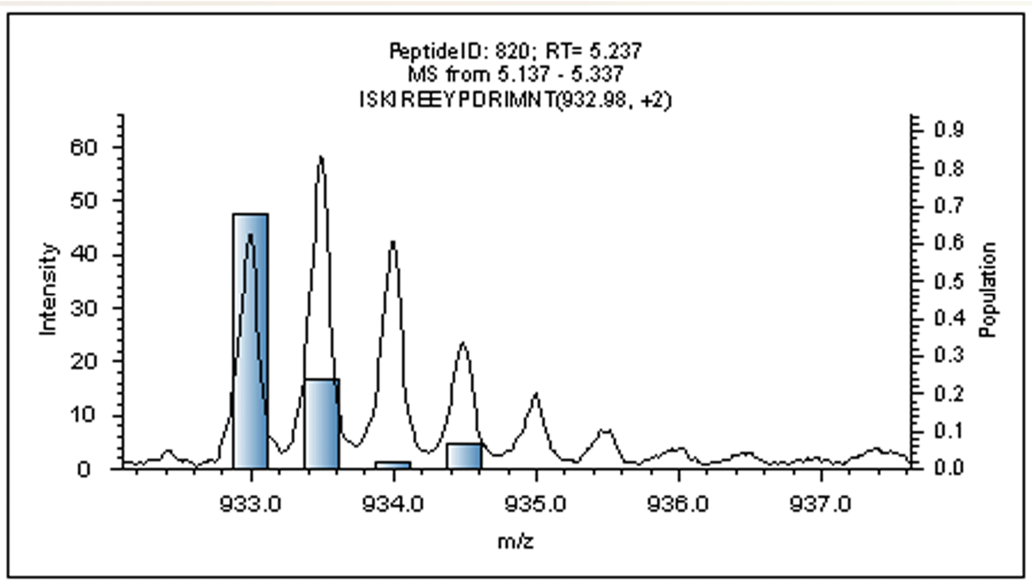

Figure 2 - Deuteration distribution being used to show an anomoly in the isotopic envelope.

The deuteration distribution is shown as a blue bar graph overlaying the mass spectrum. The fourth bar of the distribution is significantly higher than the third, suggesting that the mass spectrum either contains chemical interference or is deviating from the expected binomial distribution.

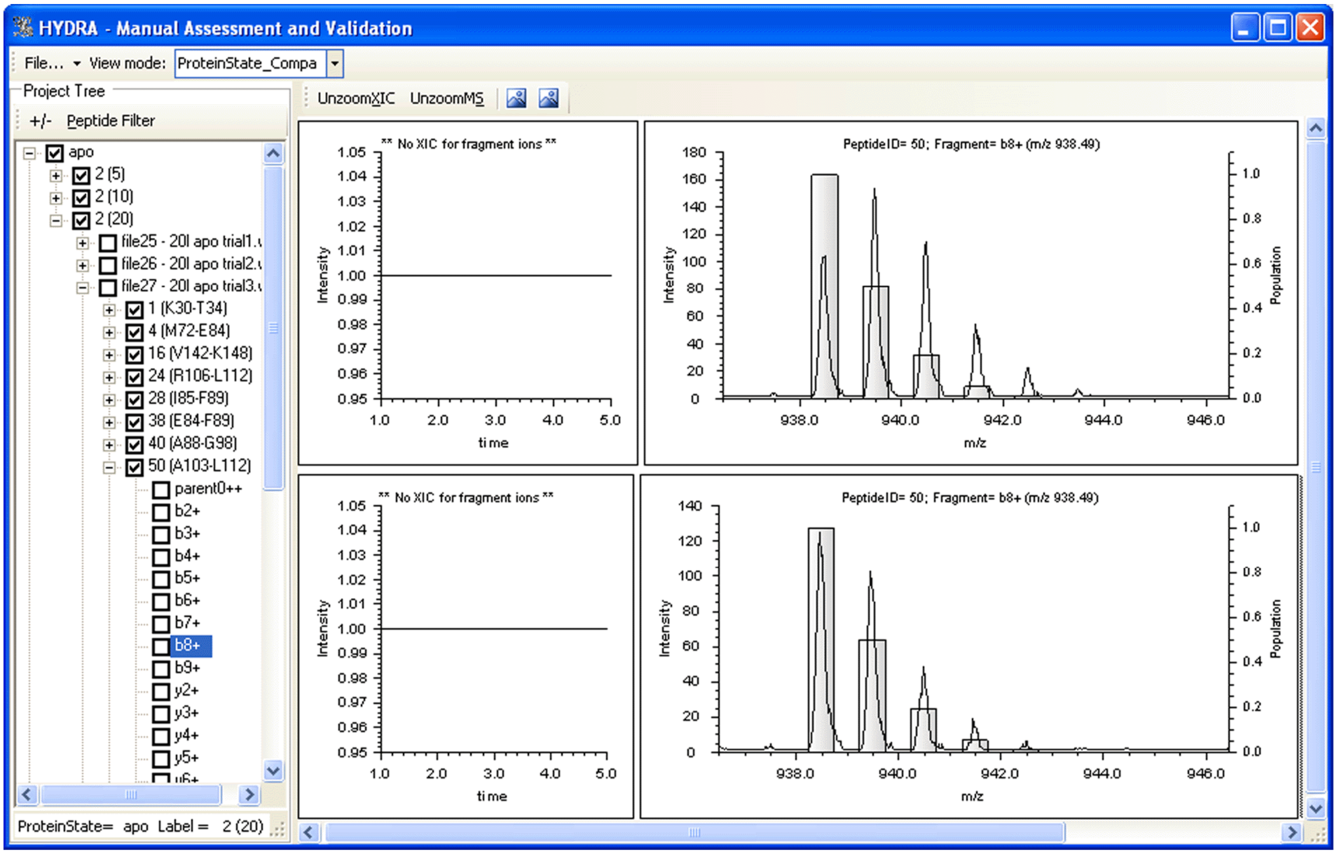

Figure 3 - Comparing two protein states at the MS/MS level

Hydra was used to extract MS/MS-level data from several peptides of calmodulin in calcium-deficient (apo) and –loaded (holo) states. The top MS shows fragment ion b_8^+ of peptide A103-L112 of apo-calmodulin while the lower MS shows the fragment ion from the holo form. Vertical alignment allows the researcher to quickly observe any differences, and this is further assisted by overlaying the theoretical unlabelled isotopic profile (clear bars).
